# The Primary Irradiation Damage of Hydrogen-Accumulated Nickel: An Atomistic Study

**DOI:** 10.3390/ma16124296

**Published:** 2023-06-09

**Authors:** Xiaoting Yuan, Hai Huang, Yinghui Zhong, Bin Cai, Zhongxia Liu, Qing Peng

**Affiliations:** 1 Key Laboratory of Material Physics of Ministry of Education, School of Physics and Microelectronics, Zhengzhou University, Zhengzhou 450052, China; 2 State Key Laboratory of Nonlinear Mechanics, Institute of Mechanics, Chinese Academy of Sciences, Beijing 100190, China; 3 School of Engineering Sciences, University of Chinese Academy of Sciences, Beijing 100049, China; 4 School of Science, Harbin Institute of Technology, Shenzhen 518055, China

**Keywords:** nickel-based alloys, hydrogen embrittlement, displacement cascades, hydrogen clusters, molecular dynamics

## Abstract

Nickel-based alloys have demonstrated significant promise as structural materials for Gen-IV nuclear reactors. However, the understanding of the interaction mechanism between the defects resulting from displacement cascades and solute hydrogen during irradiation remains limited. This study aims to investigate the interaction between irradiation-induced point defects and solute hydrogen on nickel under diverse conditions using molecular dynamics simulations. In particular, the effects of solute hydrogen concentrations, cascade energies, and temperatures are explored. The results show a pronounced correlation between these defects and hydrogen atoms, which form clusters with varying hydrogen concentrations. With increasing the energy of a primary knock-on atom (PKA), the number of surviving self-interstitial atoms (SIAs) also increases. Notably, at low PKA energies, solute hydrogen atoms impede the clustering and formation of SIAs, while at high energies, they promote such clustering. The impact of low simulation temperatures on defects and hydrogen clustering is relatively minor. High temperature has a more obvious effect on the formation of clusters. This atomistic investigation offers valuable insights into the interaction between hydrogen and defects in irradiated environments, thereby informing material design considerations for next-generation nuclear reactors.

## 1. Introduction

The diffusion and retention of hydrogen (H) in high-strength metallic materials typically exert a detrimental influence on their mechanical properties, ultimately leading to material failure. This phenomenon is commonly known as hydrogen embrittlement (HE) [1,2,3]. Two major mechanisms have recently been proposed to elucidate this phenomenon: hydrogen-enhanced decohesion (HEDE) and hydrogen-enhanced localized plasticity (HELP). The HEDE mechanism posits that H atoms weaken the cohesion strength between metal atoms, resulting in the creation of new surfaces along cracks or grain boundaries. Consequently, this process leads to the brittle fracture of materials [4,5,6]. On the other hand, the HELP mechanism suggests that defects such as dislocations and grain boundaries act as H atom traps, leading to the formation of H clusters or H-dislocation complexes. These H-related complexes, in turn, facilitate dislocation nucleation and migration, ultimately causing localized plastic deformation in the materials [7,8].

Nickel-based alloys belong to a category of high-strength metallic materials renowned for their outstanding mechanical properties and high-temperature oxidation resistance, rendering them highly promising as structural materials for Gen-IV nuclear reactors [9,10,11]. However, when exposed to reactor environments, these alloys undergo erosion by coolants (e.g., H_2_O → 2H^+^ + OH^−^) and nuclear transmutation reactions (e.g., ^58^Ni + n → ^59^Ni + γ, ^59^Ni + n → ^59^Co + H) due to neutron irradiation [12]. Consequently, the segregation and diffusion of H atoms within the lattice structures of the alloys occur. Moreover, the bombardment of high-energy neutrons leads to the generation of various point defects (i.e., vacancies and self-interstitial atoms (SIAs)), which tend to aggregate into clusters such as voids and dislocation loops.

In recent years, a considerable amount of research has been dedicated to examining the formation of H clusters under various conditions. For instance, Harada et al. [13] conducted a study that revealed the significant influence of H concentration on the formation of H-vacancy clusters in Ni–H systems. Similarly, Kuhr et al. [14] demonstrated that the H atoms at grain boundaries tend to exist either as isolated atoms or as clusters comprising only a few H atoms. While there is a substantial body of research on the formation mechanism and vacancy trapping of H in Fe and W, fewer studies have explored the damage behavior of the Ni matrix resulting from the synergistic interplay of H and point defects during cluster formation in irradiation environments. Lu et al. [15,16] carried out a systematic investigation into the behavior of H and/or He in single-crystal W and at its grain boundaries, proposing the vacancy capture mechanism for H bubble nucleation and growth. Becquart et al. [17] employed a first-principles approach to examine the properties of the point defects, as well as impurity atoms, in metallic W and Fe, uncovering the interaction mechanism on a vacancy with H/He. Serra et al. [18] focused on investigating the impact of defect traps on deuterium permeation in RAFM F82H alloys, while Hayward et al. [19] employed the functional density theory to calculate the interplay between α-Fe vacancies and H. These studies explored the roles of the defects and transmutation gas elements produced in different metals [17,18,19,20,21]. Nevertheless, limited research has been conducted on the synergistic effect of irradiation-induced defects and impurity H to elucidate the mechanism underlying HE failure in Ni alloys.

In this study, the HE mechanism of Ni alloys within an irradiation environment is investigated; in particular, the influences of the H concentration, cascade energy, and irradiation temperature on the interaction between the H atoms and irradiation-induced defects in metallic Ni are explored. To achieve this, a molecular dynamics (MD) approach is employed, focusing on the analysis of these irradiation-induced defects and the identification and distribution of H-related clusters. The findings of this study offer crucial insights into the comprehension and analyses of the nickel-based alloys utilized in Gen-IV nuclear reactors, as well as advance our understanding of irradiation damage in nickel-based alloys.

## 2. Simulation Methodology

The MD simulations were conducted utilizing the large-scale atomic/molecular massively parallel simulator code [22]. The OVITO 3.8.0 software [23] was employed for visualizing the calculation results. The interactions among the Ni atoms were described using the embedded atom method (EAM) potential developed by Bonny et al. [24], which is known for its accurate depiction of Ni defects. For the H–Ni and H–H interactions, the Beck potential [9] was adopted, which has been demonstrated by Torres et al. [9,25] to effectively model the formation energy of H defects. To simulate a displacement cascade, each potential was combined with a Ziegler–Biersack–Littmark (ZBL) potential [26].

**Figure 1:**
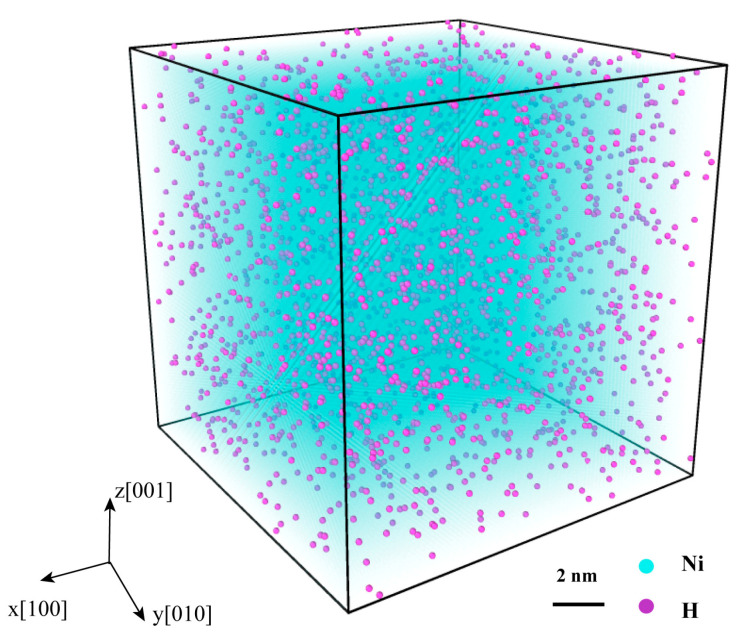
Single-crystal Ni model inserted H atoms with a certain concentration, in which the blue and purple spheres represent Ni and H atoms, respectively.

Initially, a single-crystal Ni system measuring 14.0 × 14.0 × 14.0 nm^3^ (consisting of 256,000 Ni atoms) was generated. Subsequently, H atoms with certain concentrations were randomly inserted into the system (see Figure 1). To attain equilibrium, a conjugate gradient energy minimization was performed, followed by a relaxation of the Ni–H system using an isothermal-isobaric (NPT) ensemble for 10 ps, until a steady state was achieved.

To ensure periodicity, periodic boundary conditions were applied in all three directions throughout each simulation. Next, a Ni primary knock-on atom (PKA) was randomly selected and directed along the <135> direction to avoid channeling. During the displacement cascades, atoms within the outermost 7 Å thickness of the box were constrained to release internal heat using a Nose–Hoover thermostat (NVT ensemble), while the remaining atoms were allowed to move adiabatically using an NVE ensemble. This process was carried out for a duration of 22 ps. Thereafter, the effects of three factors were investigated according to the following settings. For the first condition, the H concentrations were set at 0, 1000, 5000, 10,000, 20,000, or 30,000 appm, corresponding to 0, 0.1%, 0.5%, 1%, 2%, or 3%, respectively. The PKA energy was fixed at 5 keV and the thermostat temperature was set to 100 K. For the second condition, the PKA energy was varied at 0.5, 1.0, 3.0, 5.0, or 10.0 keV, while maintaining a constant thermostat temperature of 100 K and a H concentration of 10,000 appm. For the third condition, the thermostat temperature was adjusted to 100, 300, 500, 700, or 900 K, while keeping the PKA energy at 3 keV and the H concentration at 10,000 appm. The choice of each variable range in this work followed the general considerations from previous studies [27,28,29,30,31,32,33], facilitating a comparative analysis. It should be noted that each case involved 10 independent MD simulations to ensure statistical validity. The point defects in the simulation box were identified using the Wigner–Seitz cell method [34]. Furthermore, the detection of a cluster (such as SIA_m_, SIA_m_V_n_, H_m_, H_m_V_n_, SIA_m_H_n_, and SIA_m_V_n_H_k_) occurred when the Ni–Ni, H–H, and Ni–H atomic distances were within 4.87, 2.45, and 2.5 Å, respectively [9,35,36].

## 3. Results and Discussion

In order to assess the influences of different factors on the damage caused by irradiation in the Ni matrix and to explore the effect of the H atoms on Ni, the analysis focused solely on the number of SIAs within the matrix, rather than that of the vacancies. This approach was chosen as H atoms can be trapped within these vacancies during collision cascades, which does not accurately reflect the extent of Ni matrix damage. Furthermore, the interaction between SIAs and H atoms is known to be weak [37]. Therefore, a statistical analysis of the interstitial evolution over time was conducted.

### 3.1. H Concentration Effect on Displacement Damage

**Figure 2:**
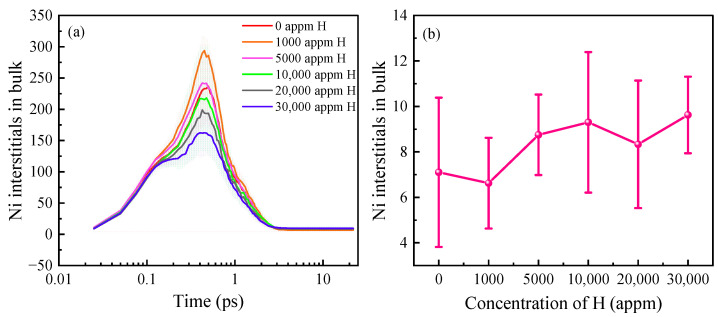
Variation in the number of SIAs with different H concentrations. (**a**) Time evolution of the number of SIAs with different H concentrations. The shaded area indicates the standard deviation of the sample means. (**b**) Number of surviving SIAs at the moment of stabilization.

To investigate the impact of the H concentrations in the Ni on the interaction between the H and irradiation-induced point defects, the PKA energy was fixed at 5 keV and the irradiation temperature was set at 100 K. The H concentrations considered ranged from 0 to 30,000 appm, corresponding to H atom numbers of 0, 256, 1280, 2560, 5120, and 7680, respectively [28]. It is common for theoretical simulations to use values larger than the actual situation [34]. The temperature was controlled at 100 K rather than other high temperatures in this simulation to avoid a coupling effect between the temperature and H concentration. Figure 2a presents the temporal evolution of the number of SIAs in the Ni system under the different H concentrations. The error bands represent the standard deviation of the mean and indicate the degree of sample dispersion. Figure 2b illustrates the remaining SIA amount in the system upon stabilization. The error bands show that the system with a H concentration of 0 appm exhibited a large deviation at the displacement spike moment. Conversely, in the H-containing system, the data deviation was minimal at a H concentration of 1000 appm and there was no significant deviation from the mean value throughout the simulation. However, as the H concentration increased to 10,000 appm, a substantial deviation from the mean value, calculated multiple times, was observed. At H concentrations from 20,000 to 30,000 appm, the deviation decreased. The degree of dispersion reflected the damage caused to the system by the different H concentrations. As demonstrated in Figure 2a, the number of SIAs exhibited temporal variation, displaying a double-peak phenomenon with a peak occurring at around 0.11 ps. A possible reason for this is that a large amount of heat was released during the cascade collision process, leading to a cascade center temperature of several thousand Kelvin [38]. At this elevated temperature, the H exhibited an extremely fast mobility and formed clusters, occupying the lattice sites and increasing the number of SIAs. The formation of these clusters impeded the annihilation of the vacancies and SIAs, leading to an increase in the number of these SIAs. This peak became more pronounced with an increase in the H concentration. At 0.5 ps, a new peak of displacement occurred, indicating that the number of defects in the cascade region reached its maximum at this moment. When the H concentration was 1000 appm, the number of SIAs at the displacement peak was higher than that in pure Ni, likely due to the occupancy of the lattice sites by the H atoms, resulting in a significant number of Ni atoms leaving their original sites. As the H concentration ranged from 5000 to 30,000 appm, the number of SIAs gradually decreased at the displacement spike. A previous study [28] suggested that, at higher H concentrations and lower temperatures, H diffusion promotes cluster formation, leading to a decrease in the H diffusion rate. Figure 2b illustrates that, during stabilization, the presence of H atoms led to an increase in the total number of surviving defects within the system, indicating an increase in the number of vacancies. Notably, at a H concentration of 1000 appm, the number of point defects was lower than that in pure Ni. This disparity could be attributed to the relatively low H concentration and the large size of the model, which hampered the displacement of the Ni atoms from the lattice sites when H atoms occupied the interstitial positions. Moreover, higher H concentrations impeded the diffusion of H, causing the rapid clustering of H atoms during the cascade collision. Consequently, as the H concentration rose, the H atoms hindered the displacement of the Ni atoms from their lattice sites, resulting in a gradual decrease in the number of SIAs at the displacement spike. In a study by Hasan et al. [28], the H diffusion in α-Fe was examined, revealing that the diffusion rate of the H was influenced by other interstitial H atoms and was higher at a H content of 0.01–0.1% compared to 1–5%.

**Figure 3:**
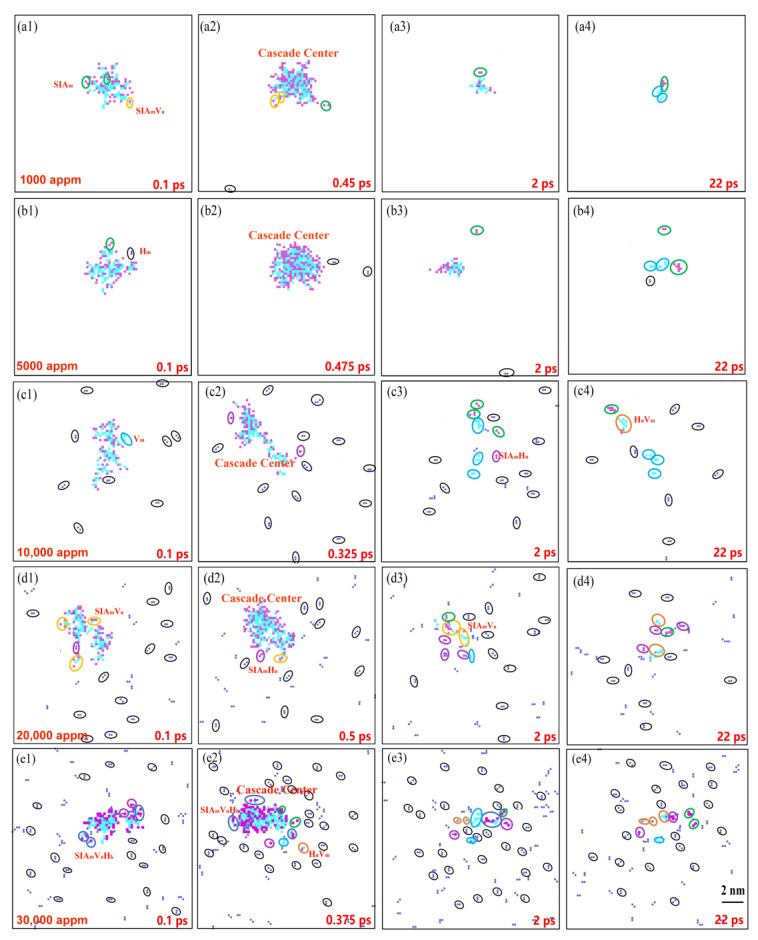
Cluster distribution at four distinct moments with different concentrations. (**a1**–**e1**) 0.1 ps. (**a2**–**e2**) Displacement spike moment. (**a3**–**e3**) 2 ps. (**a4**–**e4**) 22 ps. (Purple, blue, and dark blue spheres indicate SIAs, vacancies, and H interstitials. Black, green, light blue, yellow, purple, orange, and dark blue circles represent H_m_, SIA_m_, V_m_, SIA_m_V_n_, SIA_m_H_n_, H_n_V_m_, and SIA_m_V_n_H_k_).

To examine the impact of H-induced damage on Ni, the distribution of clusters in the Ni model was analyzed further. Figure 3 presents the cluster distributions at the different H concentrations, focusing on four distinct moments: 0.1 ps (i.e., the ballistic phase), the displacement spike, 2 ps (i.e., the annealing phase), and 22 ps (i.e., the stabilization moment). Upon combining Figure 2a with Figure 3, it can be inferred that the H concentration did not significantly affect the time required to reach the displacement peak. While the probability of H clustering increased with a higher concentration, there was no significant distinction in the clustering of atoms at the cascade center for a specific temperature and PKA energy. Given the strong correlation between the cascade collisions and PKA energy, the number of H atoms within the cascade center could exceed 30 at the displacement spike moment. At a concentration of 1000 appm, the cluster distributions at the four moments (see Figure 3(a1–a4)) indirectly indicated a lower H clustering probability. This observation aligns with the findings in Figure 2 for a H concentration of 1000 appm. In comparison to the other systems, the Ni model with a H concentration of 1000 appm neither formed clusters nor impeded atom movement, resulting in the fastest H diffusion rate. Consequently, at low H concentrations, interstitial H atoms promoted the formation of Frenkel pairs, leading to a lower number of SIAs compared to that of pure Ni. However, at higher concentrations, the H interstitials aggregated into clusters and occupied the vacancy sites, inhibiting the recombination of the SIAs and vacancies, thus increasing the number of defects during stabilization. This observation is further supported by the number of SIAs at the stabilization moment in Figure 2b. The cluster distribution reveals an increase in H clusters with concentration, elucidating the decrease in the number of vacancies with an increasing H concentration. This effect can be attributed to the formation of H-related clusters in the system. The cluster distributions at all the stages and concentrations predominantly exhibited small H clusters, which is consistent with the findings of Kuhr et al. [14]. Additionally, due to the inability of H atoms to form large H–H clusters alone, self-trapping becomes challenging to achieve, favoring H_2_V complexes, as has been indicated by previous studies [21,39]. At the stabilization moment, the number of H-related clusters gradually increased with the concentration. Consequently, the presence of H atoms significantly influenced the formation of the final defects, highlighting the substantial impact of H interstitials on the irradiation damage of Ni during cascade collisions. Furthermore, different H concentrations yielded varying effects on the defect formation, with the concentration factor playing a crucial role, particularly in the generation of small H clusters.

### 3.2. PKA Energy Effect on Displacement Damage

**Figure 4:**
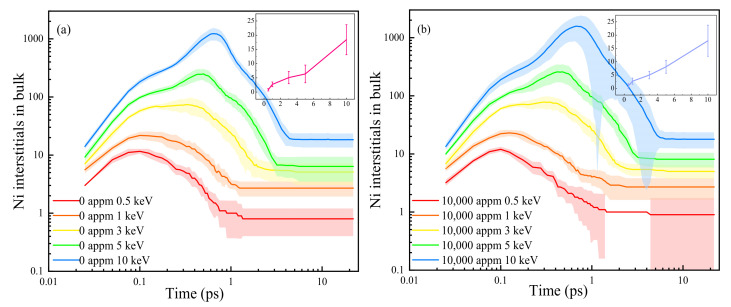
Time evolution of the number of SIAs produced in pure Ni (**a**), and H-containing Ni (**b**) systems with different PKA energies, in which the number of final stable defects is shown in the inset. H concentration is set to 10,000 appm in panel (**b**).

Figure 4 illustrates the typical time evolution of the number of SIAs during the displacement cascades at different PKA energies in the pure Ni and H-containing Ni systems. As the PKA energy increased, the peak value and time to reach the peak of the SIAs also increased, along with the number of final surviving SIAs in both the H-containing system and pure Ni. A longer duration of the thermal spike prolonged the time required to reach stability. Comparing the system with a H concentration of 10,000 appm to the pure Ni, the number of SIAs exhibited consistent trends over time. At lower PKA energies, there were no significant differences in the SIA evolutionary processes or the number of SIAs at the stabilization moment between the two systems (see Figure 4). However, at a PKA energy of 10 keV, the system with a H concentration of 10,000 appm exhibited a significantly higher number of SIAs compared to that of the pure Ni. Conversely, the error band was wider in the Ni system with a H concentration of 10,000 appm. Both systems showed larger errors at lower energies and smaller errors at higher energies. During the stabilization phase, both systems experienced increased errors and the presence of H atoms affected the number of surviving defects. Figure 4b demonstrates that, at E_PKA_ = 10 keV, the number of SIAs in the H-containing system exhibited irregular error data during the annealing phase. It is important to note that the presence of H had a significant impact on the simulation data for the vacancies and SIAs, leading to substantial deviations from standard quantities. At E_PKA_ = 0.5 keV, no error occurred between 1.45 and 4 ps due to the low PKA energy, which annihilated the final vacancies with SIAs in the partial cascade collision process, resulting in no discrepancy between the simulated and standard data (both being one). However, the Frenkel pairs formed during the latter part of the simulation process were not yet fully compounded, resulting in data fluctuations. After stabilization, the error between the simulated and normalized values persisted, but the values remained unchanged. Oudriss et al. [40,41] demonstrated that the presence of H in metallic Ni promotes the formation of vacancies. The insets in Figure 4 illustrate the numbers of the surviving SIAs in the systems, indicating that the final number of surviving SIAs was more dependent on the energetic effect, and that the presence of H impacted the final number of defects. The trend in the number of interstitials at the stabilization stage aligned with the Norgett–Robinson–Torrens (NRT) model [42]. All these results are consistent with observations from other studies [31,35,43,44].

**Figure 5:**
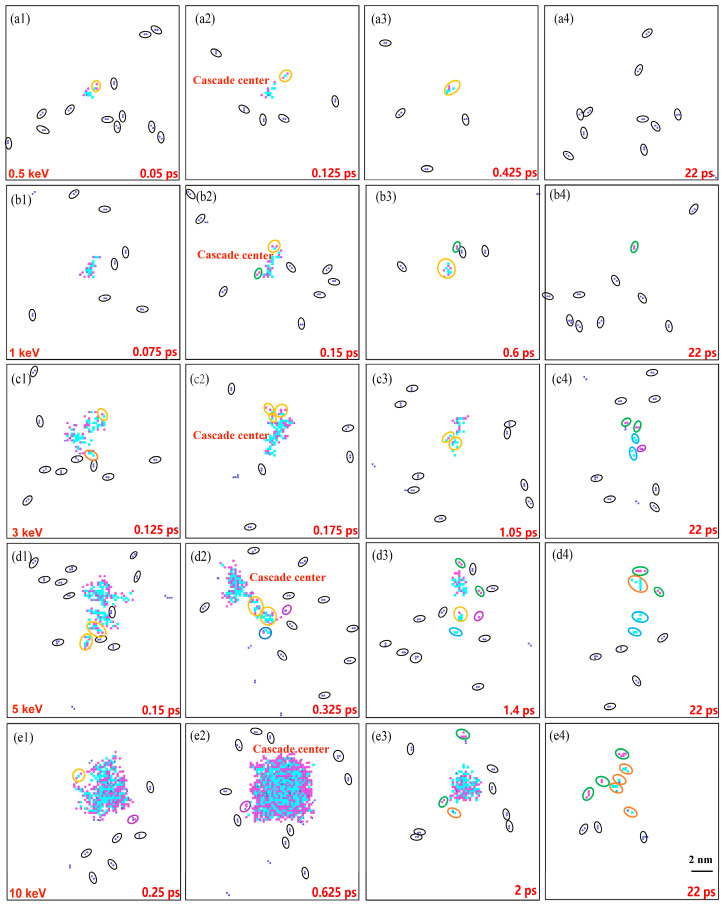
Cluster distributions at different moments with different PKA energies. (**a1**–**e1**) 0.1 ps. (**a2**–**e2**) Displacement spike moment. (**a3**–**e3**) 2 ps. (**a4**–**e4**) 22 ps. The identification of different atoms and defects is similar to the legend in Figure 3.

**Figure 6:**
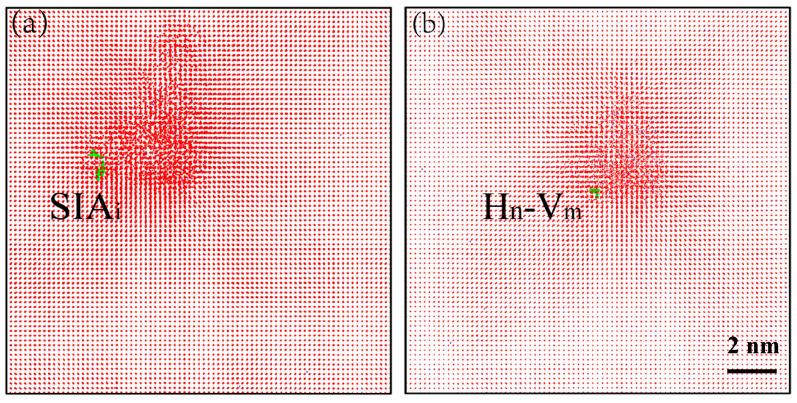
Cluster trajectory formed at PKA energy of 5 keV. (**a**) Trajectory of a SIA cluster. (**b**) Trajectory of a vacancy-hydrogen cluster. The green line indicates the route of cluster motion.

To gain a comprehensive understanding of the influence of the PKA energy on the irradiation damage, the defect clustering and distributions at different moments are shown in Figure 5. The number of H clusters remained relatively stable as the PKA energy increased. However, the types of clusters became more diverse at higher PKA energies, as a wide range of atoms collided at the center of the cascade. During these cascade collisions, the lattice atoms underwent displacement, resulting in a significant number of Frenkel pairs. At the peak moment, the point defects aggregated and temporarily formed large clusters around the cascade. After the vacancies and interstitials combines, the clusters tended to stabilize. Comparing the cluster distributions in the four phases, the cluster size progressively increased within the same phase as the PKA energy increased. At the ballistic stage, displacement spike moment, or annealing phase, clusters gathered at the cascade center as the number of Ni point defects increased with the increasing PKA energy. At 22 ps, the number of surviving interstitials and vacancies in the system increased with the increasing PKA energy. Consequently, the types of clusters generated by the interstitial H atoms, H_n_V_m_, SIA_m_, and SIA_m_H_n_, became more abundant, signifying the significant influence of the PKA energy on the cluster formation. The number of interstitial clusters surpassed that of the vacancy clusters, indicating a higher diffusion rate for the SIAs compared to that of the vacancies [45]. The trajectories of the clusters, which are depicted in Figure 6, reveal that the SIAs had a broader movement range compared to that of the vacancies, while the vacancies exhibited a stronger ability to trap H. These findings align with those from prior studies [37,46,47]. At 0.5 keV, the PKA energy was insufficient to cause significant damage, resulting in only small H clusters remaining in the simulation box after the stabilization. Hence, the irradiation damage was relatively low at low energies and the impact of the displacement defects on the interstitial H atoms was minimal. However, at 5 keV, the intense collisions generated by high kinetic energy caused the H atoms to aggregate. The resulting cluster types in the final system included H atoms trapped by vacancies, impeding the annihilation of the SIAs with the vacancies, while promoting the formation of SIA clusters.

### 3.3. Simulation Temperature Effects on Displacement Damage

**Figure 7:**
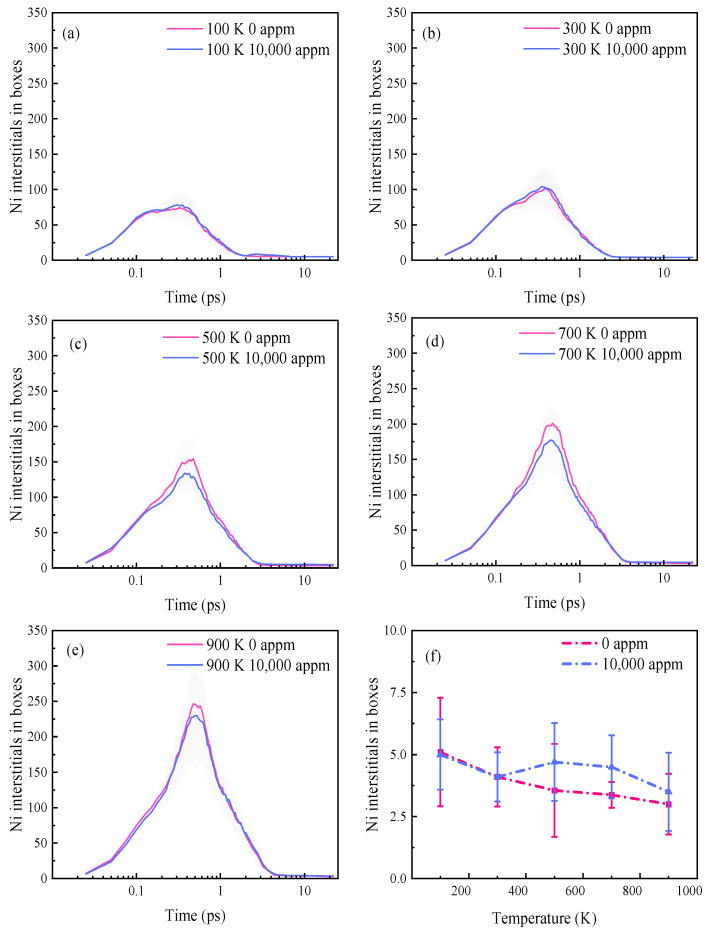
Time evolution of the number of SIAs in pure Ni and H-containing Ni systems at different temperatures. (**a**) 100 K. (**b**) 300 K. (**c**) 500 K. (**d**) 700 K. (**e**) 900 K. (**f**) Number of stable SIAs as a function of temperature.

Figure 7a–e illustrate the temporal evolution of the number of SIAs at different temperatures. These trends align with the findings of Huang et al. [31] and Béland et al. [48], in which they utilized the same potential function. The time required for the defects to reach their peak increased with the temperature, and the number of SIAs at the peak moment significantly increased. Consequently, higher temperatures facilitated defect generation during the displacement peak. The time needed for the annealing phase also lengthened with the temperature, since reaching stability took more time. Hasan et al. [28] found that the mean-squared displacements (MSDs) of H atoms in α-Fe increased linearly over time, in the temperature range from 350 to 900 K. Similarly, Torres et al. [9] simulated the behavior of interstitial H diffusion in Ni using the MD method and demonstrated that the slope of the MSD was directly proportional to the diffusion coefficient of the H atoms, within the temperature range from 400 to 900 K. This implied that the diffusion rate of H increased with the rising temperature in this range. The curvilinear relationships depicted in Figure 7a,b indicate that, at 100 and 300 K, the number of SIAs in the pure Ni throughout the evolution was more than that in the Ni system with a H concentration of 10,000 appm. At higher irradiation temperatures (500–900 K), the number of SIAs in the system with a H concentration of 10,000 appm was lower than that in the pure Ni system. This was because the H atoms exhibited reduced activity and remained relatively stationary at lower temperatures, thereby promoting vacancy generation and resulting in an increased number of SIAs. At higher temperatures, clusters formed readily due to the accelerated diffusion of the H atoms during the cascade collisions in the H-containing simulation box. The trapping effect of the vacancies on the H led to a progressive increase in the number of SIAs at the peak moment, as depicted in Figure 7c–e. Figure 7f presents the number of surviving SIAs as a function of the simulation temperature. The number of surviving SIAs decreased with the increasing temperature in the pure Ni, which is in line with previous studies [46,49]. This phenomenon can be attributed to the accelerated rate of atom migration and the annihilation of the Frenkel pairs (see Figure S2) caused by the elevated temperature. Consequently, there was a decrease in the number of defects during stabilization, promoting the annihilation of these defects. In the simulation box with a H concentration of 10,000 appm, the number of SIAs tended to increase at 500–900 K due to the enhanced mobility of the interstitial H at these temperatures [9]. As the temperature continued to rise, the formation of the H clusters influenced the rate of the H diffusion, resulting in a slight decrease in the number of SIAs at 700–900 K. Moreover, the presence of interstitial H could also impact the recombination of the Frenkel pairs to some extent.

**Figure 8:**
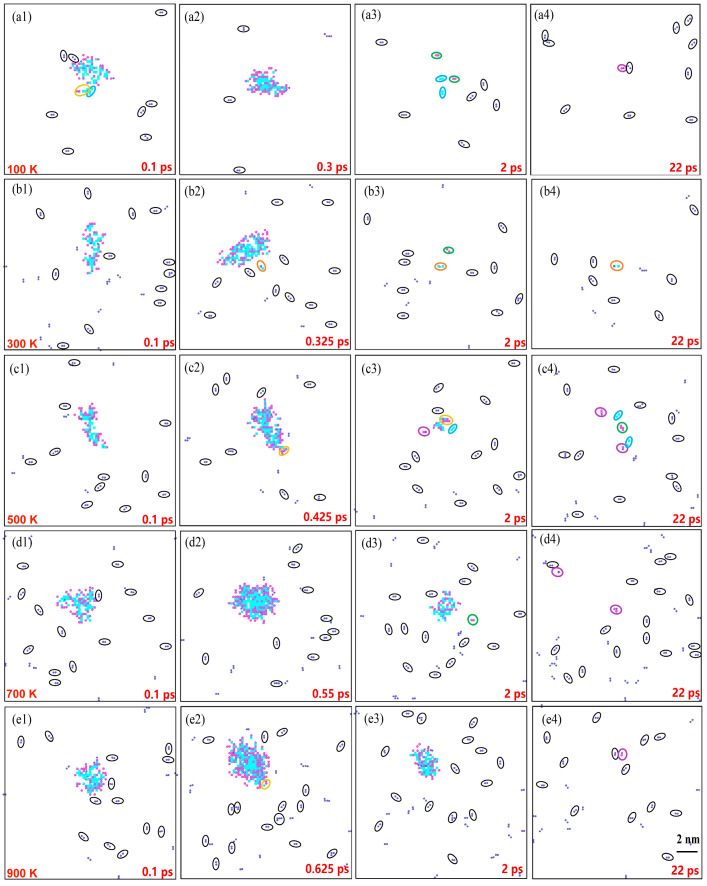
Distribution of clusters at different temperatures for four selected moments. (**a1**–**e1**) 0.1 ps. (**a2**–**e2**) Displacement spike moment. (**a3**–**e3**) 2 ps. (**a4**–**e4**) 22 ps. The identification of different atoms and defects is similar to the legend in Figure 3.

**Figure 9:**
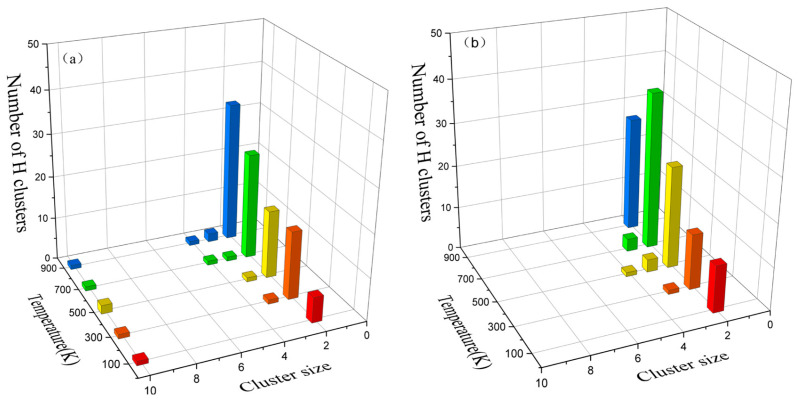
Distribution of the number of clusters at different temperatures. (**a**) Displacement spike moment. (**b**) Stabilization moment.

Figure 8 illustrates the distribution of the clusters as a function of the temperature. At 0.1 ps, the number of H clusters progressively increased with the temperature, as depicted in Figure 8(a1–e1). The number of H atoms in the clusters located at the cascade center showed an increasing trend. As the rate of H diffusion increased with the temperature, more H atoms disperses throughout the central region of the cascade. The cluster distribution at the displacement spike moment is presented in Figure 8(a2–e2), revealing a variation in the time taken to reach the peak due to the temperature. Higher temperatures corresponded to longer times to peak and larger numbers of interstitials at this peak. At 2 ps, the number of point defects decreased due to the annihilation of the vacancies with interstitials in the simulation box. However, as the temperature increased, the thermal peak duration extended, and the annealing time gradually lengthened. Consequently, at 500–900 K, the number of point defects at the center of the cascade exceeded that at lower temperatures at the same time point, as depicted in Figure 8(a3–e3). Figure 8(a4–e4) demonstrate that, during the stabilization phase, the Frenkel pairs remained as clusters formed by aggregation, which were unavailable for annihilation. At 0.1–2 ps, observations from 100 to 900 K revealed an abundance of H–H clusters in the simulation box, with clusters of other types primarily emerging around the cascade center position. With the annihilation of the vacancies and SIAs, the number of point defects gradually decreased. Clusters were formed under the influence of the H atoms, and the types of clusters increased, aligning with the study by Kuhr et al. [14]. Lu et al. [37] investigated the H diffusion behavior in bcc Fe and demonstrated that most of the binding energies of the H_m_, H_m_V_n_, or SIA_m_H_n_ clusters were positive with a small value when m < 6, so it was barely possible to form H clusters via self-trapping. This suggested the presence of H as small clusters, which was also demonstrated by the distributions of the cluster counts at the displacement spike and stabilization moments, as shown in Figure 9. In Figure 9, although the increase in the temperature may have increased the number of the clusters, it was difficult to increase the size of the clusters.

## 4. Conclusions

In this work, MD simulations were used to investigate the interaction between the H atoms and displacement defects in metallic Ni under different conditions. The key findings can be summarized as follows:

(a) The presence of solute H atoms has an important effect on the formation of a displacement spike and, in particular, can induce the double-peak phenomenon. Due to the trapping of H atoms by vacancies, an increase in the solute H concentration can result in a higher number of surviving SIAs.
(b) The increase in the PKA energy intensified the diffusion rate of the solute H atoms, promoting the recombination of H with vacancies while reducing the annihilation of the SIAs and vacancies. This caused more SIA clusters to form with the increasing PKA energy, leaving more severe damage in the H-containing system.
(c) Although the increase in the temperature exacerbated the formation of clusters, especially H-related clusters, it was difficult to form large clusters due to the increase in the defect diffusion ability and small binding energies between the H and other clusters.

In conclusion, this work sheds light on the intricate interplay between the H atoms and displacement defects in metallic Ni, emphasizing the influence of the interstitial H concentration, PKA energy, and temperature on defect formation and clustering dynamics. All these results can provide a reliable theoretical basis and reference for the assessment of the irradiation tolerance of nickel-based alloys in nuclear applications.

## Data Availability

The data that support the findings of this study are available from the corresponding author upon reasonable request.

## Supplementary Materials

The following supporting information can be downloaded at: https://www.mdpi.com/article/10.3390/ma16124296/s1, Figure S1: The trajectories of hydrogen atoms at different temperatures. An enlarged view of the center of each temperature cascade is attached to the figure. The red atoms indicate nickel atoms, the green lines indicate the trajectories of hydrogen atoms, and the blue atoms indicate hydrogen atoms; Figure S2: Number of defects at displacement peak moments at different temperatures.
