# Supplementary material for: The Primary Irradiation Damage of Hydrogen-Accumulated Nickel: An Atomistic Study
Source: Materials (Basel). 2023 Jun 9;16(12):4296. doi: 10.3390/ma16124296 (PMC10304727; doi:10.3390/ma16124296)
Supplement: Supplementary file 1 [file materials-16-04296-s001.zip › materials-2409758-supplementary.pdf]

Supplementary Information

1. Hydrogen trajectories at different temperatures

In the present work, the molecular dynamics approach is applied to the simulations and the potential functions between Ni–H and H–H are used as in Torres et al. The potential function plays an important role in the simulations, and different potential functions give different results in the simulations. The collision process in this simulation lasted 22 ps, and it is known from the literature [1] that the displacement amplitude of hydrogen atoms is not particularly large during this time period, but the displacement distance gradually increases with the increase of temperature, as shown in Fig. S1. Therefore, when studying different concentrations and different energy factors in this paper, a temperature of 100 K is chosen, when the motion of hydrogen atoms mainly relies on cascade collisions to obtain kinetic energy to affect the substrate.

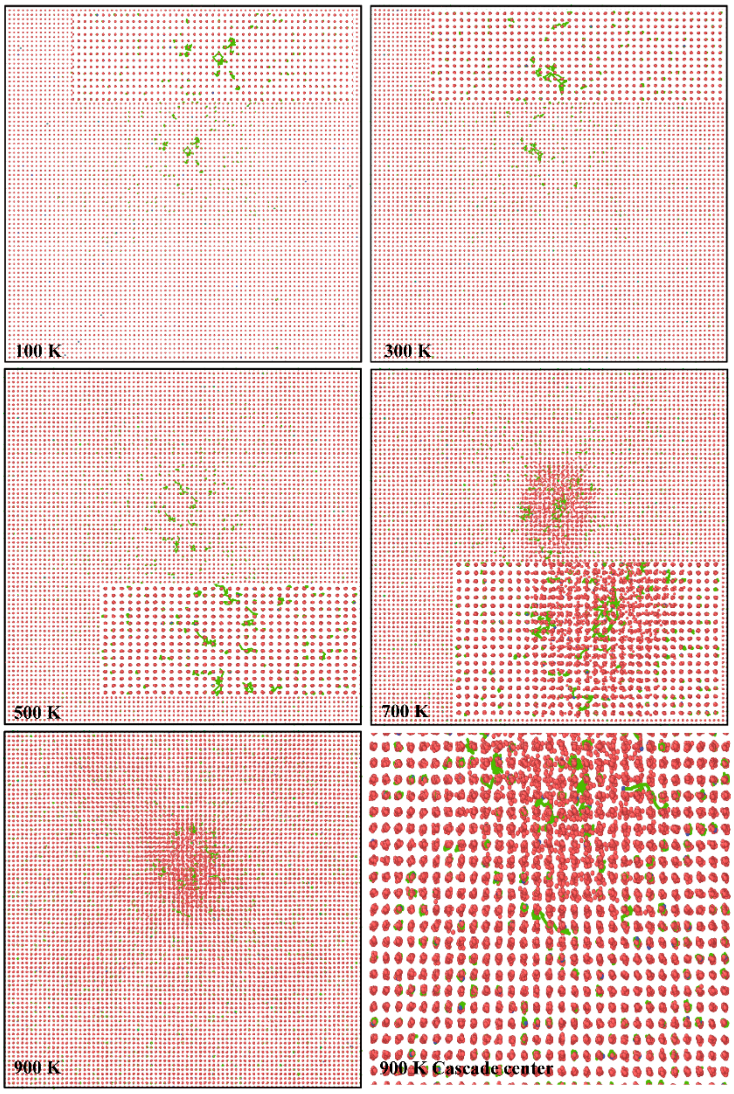

Figure S1. The trajectories of hydrogen atoms at different temperatures. An enlarged view of the center of each temperature cascade is attached to the figure. The red atoms indicate nickel atoms, the green lines indicate the trajectories of hydrogen atoms, and the blue atoms indicate hydrogen atoms.

The variation of peak moment defects at different temperatures is shown in Fig. S2. As can be seen from the figure, the number of defects at the peak moment increases gradually as the temperature increases. It can be seen that the high temperature promotes the generation of defects at the peak moment.

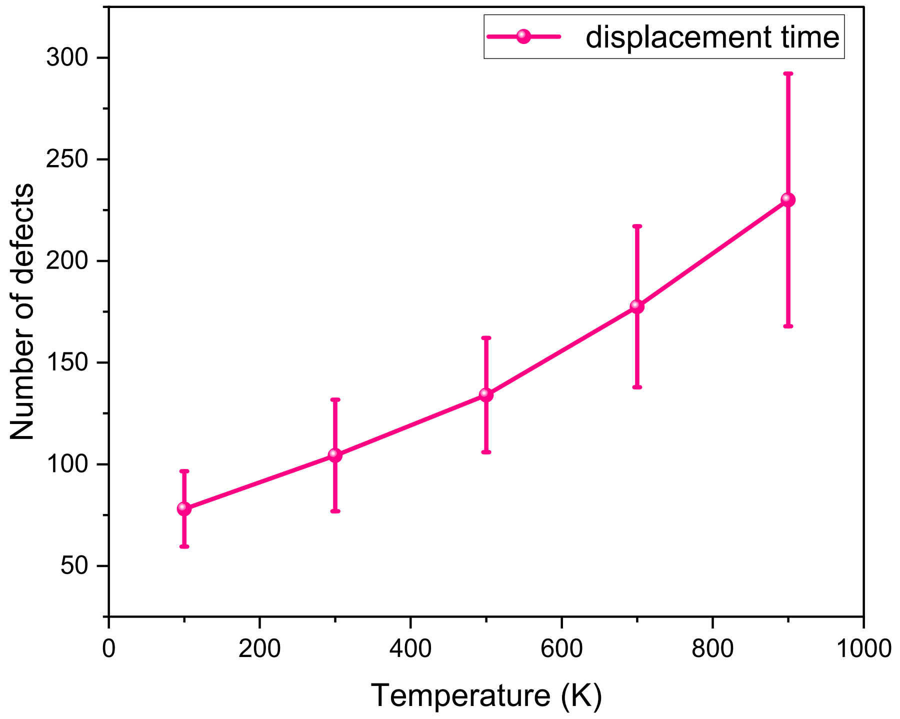

Figure S2. Number of defects at displacement peak moments at different temperatures.

Supplementary Reference

[1] E. Torres, J. Pencer, D.D. Radford. Atomistic simulation study of the hydrogen diffusion in nickel, Comp. Mater. Sci., 152 (2018): 374-380, https://doi.org/10.1016/j.commatsci.2018.06.002.
